# Acute and chronic safety and efficacy of dose dependent creatine nitrate supplementation and exercise performance

**DOI:** 10.1186/s12970-016-0124-0

**Published:** 2016-03-31

**Authors:** Elfego Galvan, Dillon K. Walker, Sunday Y. Simbo, Ryan Dalton, Kyle Levers, Abigail O’Connor, Chelsea Goodenough, Nicholas D. Barringer, Mike Greenwood, Christopher Rasmussen, Stephen B. Smith, Steven E. Riechman, James D. Fluckey, Peter S. Murano, Conrad P. Earnest, Richard B. Kreider

**Affiliations:** Department of Health and Kinesiology, Exercise and Sport Nutrition Laboratory, Texas A&M University, College Station, TX 77843-4243 USA; Department of Health and Kinesiology, Center for Translational Research in Aging and Longevity, Texas A&M University, College Station, TX 77843-4243 USA; United States Military-Baylor University Graduate Program in Nutrition, Joint Base, San Antonio, TX 78234 USA; Department of Animal Science, Texas A&M University, College Station, TX 77843-4243 USA; Department of Health and Kinesiology, Human Countermeasures Laboratory, Texas A&M University, College Station, TX 77843-4243 USA; Department of Health and Kinesiology, Muscle Biology Laboratory, Texas A&M University, College Station, TX 77843-4243 USA; Department of Nutrition and Food Science, Texas A&M University, College Station, TX 77843-4243 USA; Nutrabolt, Bryan, TX 77807 USA

**Keywords:** Creatine, Nitrate, Creatine nitrate, Nutrition, Supplementation, Exercise performance

## Abstract

**Background:**

Creatine monohydrate (CrM) and nitrate are popular supplements for improving exercise performance; yet have not been investigated in combination. We performed two studies to determine the safety and exercise performance-characteristics of creatine nitrate (CrN) supplementation.

**Methods:**

Study 1 participants (*N* = 13) ingested 1.5 g CrN (CrN-Low), 3 g CrN (CrN-High), 5 g CrM or a placebo in a randomized, crossover study (7d washout) to determine supplement safety (hepatorenal and muscle enzymes, heart rate, blood pressure and side effects) measured at time-0 (unsupplemented), 30-min, and then hourly for 5-h post-ingestion. Study 2 participants (*N* = 48) received the same CrN treatments vs. 3 g CrM in a randomized, double-blind, 28d trial inclusive of a 7-d interim testing period and loading sequence (4 servings/d). Day-7 and d-28 measured Tendo™ bench press performance, Wingate testing and a 6x6-s bicycle ergometer sprint. Data were analyzed using a GLM and results are reported as mean ± SD or mean change ± 95 % CI.

**Results:**

In both studies we observed several significant, yet stochastic changes in blood markers that were not indicative of potential harm or consistent for any treatment group. Equally, all treatment groups reported a similar number of minimal side effects. In Study 2, there was a significant increase in plasma nitrates for both CrN groups by d-7, subsequently abating by d-28. Muscle creatine increased significantly by d-7 in the CrM and CrN-High groups, but then decreased by d-28 for CrN-High. By d-28, there were significant increases in bench press lifting volume (kg) for all groups (PLA, 126.6, 95 % CI 26.3, 226.8; CrM, 194.1, 95 % CI 89.0, 299.2; CrN-Low, 118.3, 95 % CI 26.1, 210.5; CrN-High, 267.2, 95 % CI 175.0, 359.4, kg). Only the CrN-High group was significantly greater than PLA (*p* < 0.05). Similar findings were observed for bench press peak power (PLA, 59.0, 95 % CI 4.5, 113.4; CrM, 68.6, 95 % CI 11.4, 125.8; CrN-Low, 40.9, 95 % CI −9.2, 91.0; CrN-High, 60.9, 95 % CI 10.8, 111.1, W) and average power.

**Conclusions:**

Creatine nitrate delivered at 3 g was well-tolerated, demonstrated similar performance benefits to 3 g CrM, in addition, within the confines of this study, there were no safety concerns.

## Background

Numerous nutrition supplements have been investigated to determine their ergogenic benefits related to exercise performance. Two supplements that are currently popular in the marketplace are creatine and nitrate. Creatine has been extensively researched and has been the subject of numerous reviews and position statements attesting to its efficacy and safety in sport and in health [5, 7, 8]. It has been well established that supplementation with creatine can increase strength performance and improve overall body anthropometry via a reduction in percent body fat and increased fat-free mass [5]. Recently, dietary nitrate ingestion has gained in popularity as a means of improving endurance performance.

Inorganic nitrate (NO_3_^−^) is an ion exhibiting limited synthesis in the body, therefore usually obtained from the diet via green leafy vegetables, while nitrites (NO_2_^−^) are also found in food as processing additives, but to a much lesser degree [13]. The ingestion of nitrate have demonstrated its importance to health in that nitrate and nitrite can be reduced to nitric oxide, which has shown to have numerous physiologic effects in exercise and health [16, 17, 19]. While extensive reviews are available elsewhere, nitrate ingestion has been shown to reduce the oxygen cost of exercise and improve exercise tolerance [22, 24]. For example, Larsen et al., reported a reduction in maximal oxygen consumption; yet a trend for improvement in time-to-exhaustion accompanying the ingestion of sodium nitrate intake at 0.1 mmol/kg/day for three days [22]. In a similar study by the same group, investigators reported a significant reduction in oxygen consumption and improvement in gross efficiency at sub-maximal workloads using the same ingestion schema [24]. Bescos et al., (2011) investigated the impact of sodium nitrate in highly trained cyclist and triathletes and found that the consumption of 10 mg/kg prior to a cycle ergometer test reduced VO_2peak_ without influencing time to exhaustion or maximal power output [3]. Further research has elaborated on these findings, showing a reduction in the amplitude of the VO_2_ slow component; hence an improvement in muscle efficiency associated with nitrate ingestion [2, 18].

Despite the number of studies associated with endurance performance, a dearth of literature exists examining nitrate supplementation on strength performance; and no studies that we are aware of have examined the role of creatine and nitrate in combination. However, the two nutrients have the potential to work synergistically. The primary aim of our current study was to examine the effects of creatine nitrate (CrN) in a two-phase, dose dependent study administering CrN at 1.5 g (CrN-Low) and 3 g (CrN-High). Study 1 of our trial examined the acute ingestion of the respective treatments for five hours following ingestion. Study 2 of our trial examined each respective treatment for 28 days. Each CrN condition was compared to a placebo (PLA) and creatine monohydrate (CrM) treatment condition. We hypothesized that CrN would increase exercise performance and related performance indices (e.g., peak power, mean power, total work, etc.) in a dose dependent manner and be equal in effectiveness to CrM. We further hypothesized that CrN ingestion would not adversely affect hepatorenal function or hemodynamics indices following acute and chronic ingestion.

## Methods

**Fig. 1:**
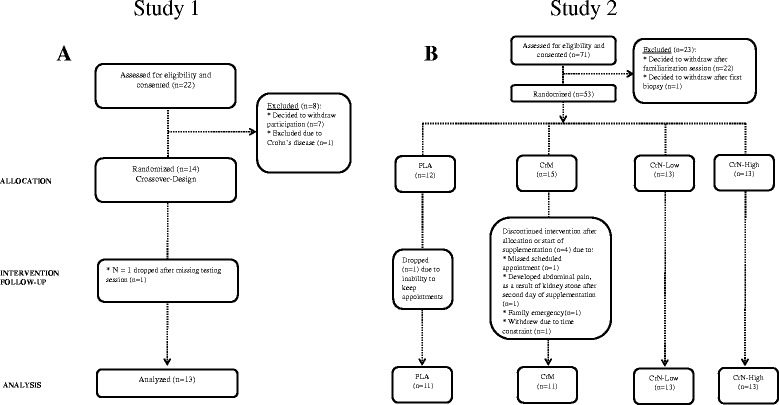
CONSORT Schematic representation of Study 1 (**a**) and Study 2 (**b**)

The current report represents studies examining the [1] acute and [2] chronic supplementation of a CrN formula. Study 1 was an acute phase study with participants ingesting each respective supplement one time in a randomized, double blind, crossover manner. Study 2 was a 28-d study using different participants receiving treatments in a randomized, double blind manner. Figure 1 presents a CONSORT schematic for Study 1 (Fig. 1a) and Study 2 (Fig. 1b). Each study was performed at the Exercise & Sport Nutrition Laboratory (ESNL) at Texas A&M University after obtaining ethical approval from the university’s ethics committee and signed informed consent from each participant. Herein we describe our overall procedures for each study followed by a detailed methodology for each test used within the studies (see below, Testing Methodology).

### STUDY ONE: acute supplementation

#### Participants

Thirteen healthy and recreationally active men (age: 22 ± 5 y, height: 177.8 ± 7.4 cm, weight: 84.1 ± 18.9 kg) were recruited to participate in Study 1. Inclusion criteria required that each participant have at least 6 mo. of resistance training immediately prior to entering the study inclusive of performing bench press and leg press or squats. Participants were excluded if they had a history of treatment for metabolic disease, hypertension, thyroid disease, arrhythmias, cardiovascular disease; currently using any prescription medication, or had ingested creatine within 6 wk of the intervention. Further exclusion criteria also included women; a history of smoking; and excessive alcohol consumption (>12 drinks/wk).

### Familiarization session

Each participant initiated Study 1 by participating in a familiarization session and was assessed for standard anthropological measurements including height, weight, blood pressure, and heart rate. Participants also completed a general health screening form that was reviewed by a registered nurse. Prior to baseline testing, each participant completed a dietary record to include three weekdays and one weekend day.

### Testing procedures

Participants were instructed to refrain from drinking alcohol, exercise, and non-steroidal anti-inflammatory drugs (NSAIDs) for 48 h before testing. Testing was initiated by obtaining participant weight, body composition measure (DXA), resting blood pressure (BP), and resting heart rate (HR). Participants were then fitted with an indwelling catheter for collection of a 20 mL blood sample at time-0 prior to supplement ingestion.

### Supplementation and assessment protocol

Upon completion of the pre-supplementation procedures, each participant was randomized in a counterbalanced manner to ingest their respective supplements providing 6.5 g total ingredients per dose. Treatments consisted of a [1] placebo (PLA: 6.5 g dextrose), [2] creatine monohydrate (CrM, 5 g creatine monohydrate, 1.5 g dextrose), [3] CrN-Low (1.5 g creatine nitrate, 5 g dextrose) or [4] CrN-High (3 g creatine nitrate, 3.5 g dextrose). All supplements were provided by Nutrabolt (Bryan, TX). Following the ingestion of each respective supplement, participants were placed into a semi-recumbent position where HR and BP were measured and blood was drawn in regular time increments of 30 min and then 1–5 h on the hour. All blood samples were analyzed for standard blood chemistries to assess hepatorenal and muscle enzyme function, as well as glucose, and blood lipids. A complete blood count with platelet differential was measured in addition to determining plasma nitrate and nitrite levels. All treatment assessments were separated by at least a 7-d washout period.

### STUDY TWO: chronic supplementation

#### Participants

Forty-eight, healthy and recreationally active males volunteered to participate in Study 2 (age: 21 ± 3 y, height: 176.8 ± 5.8 cm, weight: 77.4 ± 20.9 kg). Baseline testing followed a similar pattern to Study 1. Participants also were asked to follow a standardized resistance-training program 2 wk before their baseline testing session (d-0) that was continued throughout the supplementation period. Participants were further requested not to change their dietary intake throughout the investigation.

### Familiarization testing

During the familiarization period, testing procedures were initiated by explaining measurements for body composition measurements, blood collection, biopsies, and exercise testing. Participants also completed a 1-repetition maximum (1RM) bench press test and a practice anaerobic sprint test on a cycle ergometer. The strength test and anaerobic capacity procedures are explained in greater detail below. Strength tests were performed using a standardized isotonic lifting protocol on an Olympic bench press [21]. Participants then were scheduled to return to the laboratory and were requested to start a standardized resistance-training program 2 wk before their baseline laboratory visit. After the baseline testing session (d-0) for Study 2 participants were assigned randomly to one of the four treatment groups, providing 5.5 g total ingredients per dose. In Study 2, participants were assigned randomly to [1] PLA (5 g dextrose, 0.5 g flavoring), [2] CrM (3 g CrM, 0.5 g flavoring, 2 g dextrose), [3] CrN-Low (1.5 g CrN, 0.5 g flavoring, 3.5 g dextrose), and [4] CrN-High (3 g CrN, 0.5 g flavoring, 2 g dextrose). Exercise testing occurred on baseline (d-0) and d-28.

### Testing procedures

Participants arrived at the laboratory on the day before baseline testing, at baseline (d-0), d-7, d-27, and d-28 for a total of five laboratory visits. Percutaneous muscle biopsies from the *vastus lateralis* occurred on the day before baseline testing, d-7, and d-27 using a modified Bergstrom needle biopsy technique following standard procedures and subsequently examined for creatine concentrations [10]. Participants were requested to fast for 12 h and refrain from exercise, alcohol, and NSAIDs consumption for 48 h prior to baseline, d-7, and d-28 testing. Participants turned in their food records upon arrival to the lab at baseline, d-7, and d-28. Participants were weighed and body composition was determined via a DXA scan. Total body water was measured via bioelectrical impedance analysis (BIA). A fasted blood sample was collected after determining body composition. On d-7, participants completed the testing session with a muscle biopsy. At baseline and d-28, after the blood sample collection, participants continued with the exercise tests, which consisted of a bench press test and an anaerobic sprint test. Following their respective treatment assignment, participants were requested to ingest four doses of their respective supplements per day (at approximately 0800, 1200, 1600, and 2000 h.) for the first 7-d. Thereafter, participants ingested supplements one time per day for the remainder of the study (d 8–28). Nutrabolt (Bryan, TX) provided all of the supplements for this study.

A side effect questionnaire was completed weekly for the duration of the study. The questionnaires were completed to determine how well participants tolerated supplementation; how well participants followed the supplementation protocol; and if participants experienced any symptoms as a result of the supplement. Supplement logs and verbal confirmation were used to monitor compliance to the supplementation protocol. After completing the first performance testing session at baseline, participants were given their required supplements and written directions on how to properly ingest the supplements during the supplementation period. Participants also engaged in a standardized, 4 d/wk, split routine, encompassing upper and lower body workouts for a total of 6 wk. Training logs were completed and maintained by each participant to detail each training session workload. A training partner or fitness instructor monitored the training sessions to verify each session was completed.

### Testing methodology

#### Anaerobic capacity

After completion of the third bench press set, participants rested for ~5 min before starting the anaerobic sprint test on a Lode Excalibur Sport 925900 cycle ergometer (Lode BV, Groningen, The Netherlands). The anaerobic sprint test consisted of a 3-min warm-up comprised of pedaling at 60 – 70 rpm against a resistance of 50 W for the first minute, 75 W for the second minute, and 100 W for the third minute. The test consisted of six sprints, lasting 6-sec each, interspersed with a 30-sec rest between each repetition. Participants were then allowed to rest for 3 min before initiating a Wingate test. Participants practiced the anaerobic sprint test during the familiarization session. They were given detailed instructions and verbal encouragement to pedal as fast as possible, at an ‘all out’ pace at each sprint during the anaerobic sprint test.

During the six, 6-sec sprints and the Wingate test (30-sec sprint), each participant pedaled, all out, against a standard workload of 7.5 J/kg/rev on the Lode ergometer. To initiate the test, we instructed each individual to pedal as fast as possible, at an ‘all out’ pace prior to application of the workload and then sprint as fast as possible (all out) for the duration of each sprint. Test-to-test variability in performing repeated Wingate anaerobic capacity tests in our lab have yielded correlation coefficients of *r* = 0.98 ± 15 % for mean power. Following each test, we recorded the seat height, pedal position, and handlebar height to use for each testing session.

#### Anthropometry

Standardized anthropological testing included assessments for body mass and height on a calibrated scale (Cardinal Detecto Scale Model 8430, Webb City, MO) and body composition via DXA (excluding cranium; Hologic Inc., Waltham, MA). Test/retest reliability studies performed on male athletes with DXA yielded mean deviation for total bone miner content and total fat-free/soft tissue mass of 0.31-0.45 %, with a mean intra-class correlation of 0.985 [1].

#### Blood collection procedures

Participants provided a (8 h) fasted blood sample via venipuncture, using intravenous (IV) catheterization (BD Insyte Autoguard, Bection, Dickinson and Company, Franklin Lakes, NJ) from the antecubital vein in the forearm according to standard phlebotomy procedures. Approximately 20 mL of whole blood was collected at each time point (i.e., 0 [unsupplemented], 0.5, 1, 2, 3, 4, 5 h) in three, pre-chilled, 10-mL (18 mg K_2_ ethylene-diaminetera-acetic acid) tubes (BD Hemogard, Franklin Lakes, New Jersey). The 10-mL EDTA tubes were pre-chilled on ice and immediately placed back on ice after each blood sampling period. Two collection tubes were centrifuged at 3000 x g for 10 min at 4 °C within 3 min of collection. Plasma was extracted and stored at −80 °C for later analysis. The third collection tube was stored at 4 °C for approximately 5 h and analyzed for a complete blood count with platelet differential.

#### Blood chemistry

All blood samples were analyzed for standard blood chemistries inclusive of alkaline phosphatase (ALP), aspartate transaminase (AST), alanine transaminase (ALT), creatinine, blood urea nitrogen (BUN), creatine kinase (CK), lactate dehydrogenase (LDH), glucose, and blood lipids (total cholesterol, high density lipoprotein [HDL], low density lipoprotein [LDL], triglycerides [TG] using a Cobas® c 111 (Roche Diagnostics, Basel, Switzerland).

The Cobas® automated clinical chemistry analyzer was calibrated according to manufacturer guidelines. This analyzer has been known to be valid and reliable in previously published reports [15]. The internal quality control for the Cobas c 111 was performed using two levels of control fluids purchased from the manufacturer to calibrate acceptable SD and coefficients of variation (C_V_) values for all aforementioned assays. Samples were re-run if the observed values were outside control values and/or clinical norms according to standard procedures. Participants were also given questionnaires at each time point of the study to assess how well participants tolerated supplementation, how well participants followed the supplementation protocol, and if participants experienced any symptoms as a result of the supplementation.

A complete blood count with platelet differential (hemoglobin, hematocrit, red blood cell counts, MCV, MCH, MCHC, RDW, white blood cell counts, lymphocytes, granulocytes, and mid-range absolute count (MID) was measured using a Abbott Cell Dyn 1800 (Abbott Laboratories, Abbott Park, IL, USA) automated hematology analyzer. The internal quality control for Abbott Cell Dyn 1800 was performed using three levels of control fluids purchased from manufacturer to calibrate acceptable SD and C_V_ values for all whole blood cell parameters. Calorimetric assay kits were used to measure plasma creatine (Sigma-Aldrich, St. Louis, MO) and plasma nitrate (Cayman Chemical, Ann Arbor, MI) concentrations. These assays yielded mean C_V_ value for plasma nitrate (±3.21 %) and plasma creatine (±6.66 %) with a test/retest correlation of *r* = 0.98 and *r* = 0.99 for plasma nitrate and creatine, respectively.

#### Food frequency

A registered dietitian collected all food logs and analyzed the results using dietary analysis software (ESHA Food Processor Version 8.6, Salem, OR). Participants were asked to record all food and beverage consumption for three days and one weekend day prior to each testing session during Study 1 and prior to baseline, d-7, and d-28 during Study 2.

#### Muscle biopsies

During the study, we collected and analyzed muscle biopsy samples to assay for creatine concentration. All samples were collected while the participant was in the supine position, where the region around the biopsy site was shaved and sterilized with 3 povidone-iodine swab sticks (Professional Disposables International, Inc., Orangeburg, NY). Lidocaine HCl (1 %) was injected underneath the skin, followed by an injection through the fascia and to the epidermis using a 10 mL syringe to anesthetize the biopsy region. After approximately 5 – 10 min a small incision of about 0.5 cm was made at the biopsy site using a sterile scalpel (Aspen Surgical, Caldedonia, MI). Pressure was applied with sterile gauze after the incision was made. A 5 mm biopsy needle was inserted into the incision and into the ‘belly’ of the *vastus lateralis* muscle. Once the biopsy needle was pushed through the fascia and settled into the correct location suction was applied using 60 mL syringe, a small muscle sample was collected with the biopsy needle. The biopsy needle was in the muscle for approximately 5 – 20 sec until the procedure was completed.

After the biopsy was obtained, the sample was removed from the needle by forcing air through the syringe connected to the biopsy needle. The muscle was quickly blotted on a sterile cover sponge to remove excess blood and then snap frozen into liquid nitrogen. The sample was then stored at −80 °C for later analysis. Immediately following the biopsy procedure, pressure was applied for at least 10 min to the incision site to halt bleeding. After bleeding had stopped, steristrips (3 M Health Care, St. Paul, MN) were applied to ensure closure of the incision. A tegaderm film (3 M Health Care, St. Paul, MN) was placed over the steristrips, followed by gauze and a self-adherent pressure bandage. Participants were provided with a biopsy care kit including multiple steristrips, tegaderm films, and the contact information of the study coordinator.

#### Muscle creatine analysis

Muscle samples were analyzed using mass spectrophotometer for muscle creatine (Cr). Samples were analyzed based on methods developed by Harris and colleagues [11, 12, 14]. The previously stored muscle samples were placed in a vacuum centrifuge (Jouan RC1010 SpeedVac Concentrator, Abbott Laboratories, Abbott Park, IL) and centrifuged for approximately 4 h. Following the dehydration process, the samples were powdered using a mortar and pestle and then placed into pre-weight microcentrifuge tubes. Perchloric acid (0.5 M) and a 1 mM ethylenediaminetetraacetic acid (EDTA) solution were used to extract the muscle metabolites. The acid solution was added to microcentrifuge tubes containing the powered muscle. Microcentrifuge tubes were placed on ice for 15 min with periodic vortexing. Samples were centrifuged at 5,000 rpm for 5 min. The supernatant was transferred into a pre-weighed microcentrifuge tube. A 2.1 M KHCO_3_ solution was used to neutralize the samples. The samples were then centrifuged a second time at 5,000 rpm for 5 min. The supernatant was removed and placed into a label microcentrifuge tube and stored at −80 °C.

The samples were thawed at room temperature with periodic vortexing. Extracts were assayed for Cr in presence of 50 mM imidazole buffer, pH 7.4; 5 mM magnesium chloride; 20 mM potassium chloride; 25 μM phosphoenolpyruvate; 200 μM ATP; 45 μM NADH; 1250 U/mL lactate dehydrogenase; and 2,000 U/mL pyruvate kinase. The reagents were individually added into 1.5 mL cuvettes. The assay was then carried out using 200 μL buffer, 100 μL potassium chloride, 25 μL NADH, 20 μL ATP, 10 μL phosphoenolpyruvate, 2 μL pyruvate kinase, 2 μL lactate dehydrogenase, 150 μL water, and 100 μL of muscle extract. Changes in absorbance were recorded with a Beckman Coulter DU 7400 Diode Array Spectrophotometer (Brea, California, USA) at a wavelength of 339 nm. 20 μL of creatine kinase (25 U/mg) was added after the initial reading. The solution was read every 5 min for 20 min for post-reaction absorbance values. Test-to-test assay variability yielded mean C_V_ values for muscle creatine (±3.69 %) with a test/retest correlation of *r* = 0.946.

#### Side effects

The side effects questionnaire was completed after each post-supplementation blood sampling period during Study 1 (acute supplementation). In other words, during Study 1, a side effects questionnaire was completed at 0.5, 1, 2, 3, 4, and 5 h post-supplementation. During Study 2 (chronic supplementation), a side effects questionnaire was completed weekly, at d-7, d-14, d-21, and d-28 of supplementation. The questionnaire was completed to determine how well participants tolerated supplementation; how well participants followed the supplementation protocol; and if participants experienced any adverse symptoms during the supplementation period. Participants were asked to rank the frequency and severity of their symptoms – dizziness, headache, tachycardia, heart skipping or palpitations, shortness of breath, nervousness, blurred vision, and unusual or adverse effects. Participants were requested to rank their symptoms with 0 (none), 1 (minimal: 1-2/wk), 2 (slight: 3-4/wk), 3 (occasional: 5-6/wk), 4 (frequent: 7-8/wk), or 5 (severe: 9 or more/wk).

#### Strength testing and bench press test

Strength testing was performed on a bench press during the familiarization session when participants completed a 1-repetition maximum (1RM) using standardized isotonic Olympic bench press procedure [21]. Participants warmed-up by performing 10 repetitions at 50 % of their estimated 1RM, 5 repetitions using 70 % of their estimated 1RM, and 1 repetition using 90 % of their estimated 1RM. One RM was determined within 5, one-repetition sets following the warm-up. Our bench press procedures show low day-to-day mean coefficients of variation and high reliability in our lab (1.1 %, intra-class, *r* = 0.99). Bench press testing was performed at baseline and d-28. The bench press test, which only took place at baseline and d-28, consisted of three total sets using a 70 % 1RM load. Two sets of ten repetitions followed by one set of repetitions to failure were performed. Participants had a 2-min rest period between sets. Peak power, average power, and average velocity were measured during each repetition of the three sets with a Tendo Fitrodyne.

#### Strength training procedures

All participants were required to follow the same resistance training routine. The resistance training routine consisted of exercise 4 d/wk split into two upper and two lower body workouts per week for a total of 6 wk. The 6 wk training protocol was periodized in 3 wk increments consisting of selected exercises for the following muscle groups. Upper body training consisted of two chest exercises, two back exercises, one shoulder exercise, one biceps exercise, one triceps exercise, and one abdominal exercise. Lower body training consisted of two-quadriceps exercises, two hamstring exercises, and one calf exercise. Each exercise consisted of three sets of 10 repetitions (wk 1–3) or 8 repetitions (wk 4–6) performed with as much weight as the participant could perform per set. Individual training logs were maintained throughout the intervention and a training partner or fitness instructor confirmed completion of each training session.

#### Supplementation protocol

Participants were assigned in a double-blind and counter-balanced manner to ingest [1] PLA (5 g dextrose, 0.5 g flavoring), [2] CrM (3 g CrM, 0.5 g flavoring, 2 g dextrose), [3] CrN-Low (1.5 g CrN, 0.5 g flavoring, 3.5 g dextrose), and [4] CrN-High (3 g CrN, 0.5 g flavoring, 2 g dextrose). Participants were asked to ingest 4 doses per day (at approximately 0800, 1200, 1600, and 2000 h) during the loading phase (d-0 [after baseline testing session] through d-7). During the loading phase participants ingested a total of [1] PLA (26 g dextrose/d), [2] CrM (12 g CrM + 2 g flavoring + 8 g dextrose/d), [3] CrN-Low (6 g CrN + 2 g flavoring + 14 g dextrose/d), and [4] CrN-High (12 g CrN + 2 g flavoring + 8 g dextrose/d). Thereafter, participants ingested supplements one dose daily at 0800 (maintenance phase) for the remainder of the study (d-8 – d-28). During the maintenance phase participants ingested a daily total of [1] PLA (6.5 g dextrose), [2], CrM (3 g CrM + 0.5 g flavoring + 2 g dextrose), [3] CrN-Low (1.5 g CrN + 0.5 g flavoring + 3.5 g dextrose), [4] CrN-High (3 g CrN + 0.5 flavoring + 2 g dextrose). Participants were instructed to take the supplements at the assigned time and to take it as soon as possible if the proper timing was missed. All supplements were provided by Nutrabolt (Bryan, TX).

#### Total body water

Total body water was determined under standardized conditions using an ImpediMed DF50 bioelectrical impedance analyzer (BIA, ImpediMed, San Diego, CA, USA). Participants were stationed in a supine position with four electrodes placed on the wrists and ankles. Bioelectrical impedance analysis has been shown to be a valid method of determining total body water [29].

### Statistical analysis

#### Data analysis

Prior to initiation of the study, we ran a priori power analysis, which indicated the appropriate sample size estimates for Study 1 and Study 2. All statistical analysis was completed utilizing SPSS 22.0 (IBM Statistics, Chicago, IL). Study data were analyzed using a repeated measured multivariate analysis of variance (MANOVA). Delta and percent change values were calculated and used to determine changes from baseline, which were analyzed by repeated measures analysis of variance (ANOVA). Participant baseline demographic data were analyzed using one-way ANOVA. Overall MANOVA effects were examined as well as MANOVA univariate group effects for certain variables when significant interactions were seen. Greenhouse-Geisser univariate tests of within-subjects time and group x time effects and between-subjects univariate group effects were reported for each variable analyzed within the MANOVA model. Data were considered statistically significant when the probability of type I error was 0.05 or less and statistical trends were considered when the probability of error ranged between *p* > 0.05 to *p* < 0.10. When a significant group, treatment and/or interaction alpha level was observed, Tukey’s least significant difference post-hoc analysis was performed to determine where significance was obtained. Lastly, we performed an analysis for relevant clinical chemistries denoting lipids, glucose, muscle and hepatorenal enzyme function changes exceeding normal clinical limits concordant to supplementation for changes from (a) baseline to d-7, (b) baseline to d-28, and (c) d-7 to d-28 using chi-square analyses. Data are presented as mean ± SD, mean change ± 95 % confidence intervals as appropriate and frequency for chi-square analyses.

## Results

### Study 1: acute supplementation

Twenty-two participants were initially recruited for Study 1, and all completed consent forms, and participated in the required familiarization session. Of the original 22 participants, 13 completed Study 1 (Fig. 1). Seven participants dropped out after the familiarization session due to time constraints and one participant did not pass medical screening due to Crohn’s disease. One participant was excluded after being randomized into a treatment group due to missing scheduled testing sessions. None of the participants dropped out of the study due to side effects related to the study protocol or supplementation.

#### Plasma creatine and nitrate

**Fig. 2:**
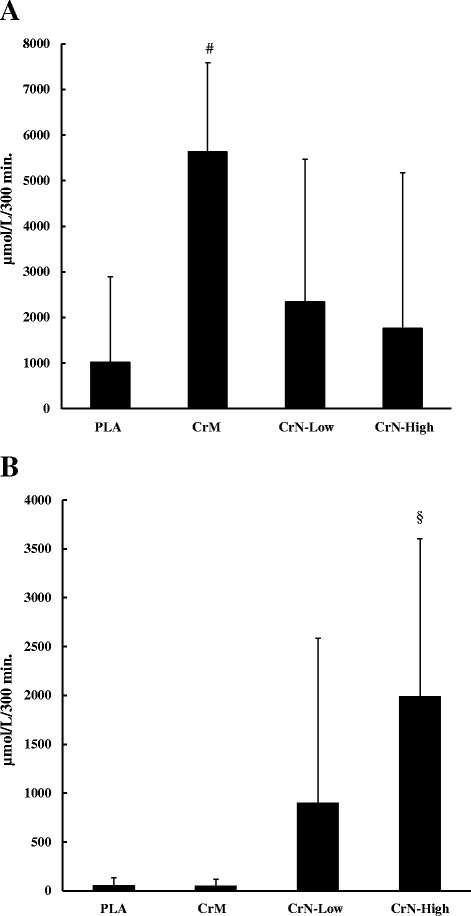
Data represent mean ± SD plasma creatine (**a**) and plasma nitrate (**b**) area-under-the-curve over 5 h post ingestion. Statistical notations (*p* < 0.05 considered significant): (#) denotes a significant difference from PLA, CrN-Low, and CrN-High. (§) denotes a significant difference from PLA and CrM

Figure 2 shows the plasma creatine area under the curve (AUC) after acute supplementation. The plasma creatine AUC for CrM (5,634.4 ± 1,949.8 μmol/L) was significantly greater than PLA (1,012.4 ± 1,882.2 μmol/L, *p* = 0.001), CrN-Low (2,342.0 ± 3,133.3 μmol/L, *p* = 0.004), and CrN-High (1,761.7 ± 3,408.8 μmol/L, *p* = 0.007). There were no significant differences in plasma creatine AUC among PLA, CrN-Low, and CrN-High. Figure 2 also shows the plasma nitrate AUC after acute supplementation. The plasma nitrate AUC for CrN-High (1,988.2 ± 1,618.8 μmol/L) was significantly greater than CrM (48.0 ± 73.1 μmol/L, *p* = 0.001) and PLA (51.4 ± 83.4 μmol/L, *p* = 0.001), but not significantly different than CrN-Low (898.8 ± 1,688.9 μmol/L, *p* = 0.10). Although there was a trend towards a greater AUC with CrN-Low there was no significant difference compared to PLA (*p* = 0.099) or CrM (*p* = 0.091).

#### Hematologic profile

Findings for participant hepatorenal muscle enzymes, and lipids are presented in Table 1. There were no significant time effects for AST (*p* = 0.12), CK (*p* = 0.06), and LDH (*p* = 0.40). However, there were significant time effects for ALP (*p* < 0.001), ALT (*p* = 0.001), BUN (*p* < 0.001), BUN: Creatinine ratio (*p* < 0.001), and glucose (*p* = 0.009). No significant group x time interactions were observed for ALP (*p* = 0.65), ALT (*p* = 0.66), AST (*p* = 0.64), CK (*p* = 0.51), LDH (*p* = 0.40), BUN (*p* = 0.76), BUN: Creatinine ratio (*p* = 0.08), and glucose (*p* = 0.12). Significant time (*p* = 0.001) and group × time effects (*p* = 0.001) were only observed for creatinine. Specifically, CrN-High had significantly greater creatinine concentration than PLA at time 0. The PLA group had significantly lower creatinine concentration than CrN-Low and CrN-High at 0.5 h. CrN-High had significantly greater creatinine concentration than CrM at 0.5 h. CrN-Low had significantly greater creatinine concentration than PLA and CrM, but significantly lower concentrations than CrN-High at 1 h. Concentrations of creatinine in CrN-High were significantly greater than PLA, CrM, and CrN-Low at 1 h and at 2 h. CrN-Low had significantly lower creatinine concentration than PLA at 2, 3, 4, and 5 h. Creatinine concentrations in CrN-High were significantly greater than PLA at 4 and 5 h. No significant time effects were observed for TC (*p* = 0.06), or the ratio (*p* = 0.09), and LDL (*p* = 0.06). While we did observe a significant time effect for HDL-C (*p* = 0.001) and TG (*p* = 0.007), no significant group × time interactions were noted for any lipid variable.

**Table 1:** Hemodynamic and hematological response to supplementation for Study 1

		Time (hours)								
	Group	0	0.5	1	2	3	4	5	Interaction	p-Level
Hemodynamics										
HR	PLA	59.7 ± 8.2	60.1 ± 7.4	58.8 ± 7.7	57.2 ± 5.3	58.0 ± 7.4	54.8 ± 6.4	56.0 ± 7.3	Group	0.23
(b/min)	CrM	63.8 ± 11.8	60.9 ± 5.7	59.9 ± 8.2	58.2 ± 7.0	56.3 ± 6.8	59.6 ± 8.8	58.5 ± 10.0	Time	0.15
	CrN-Low	60.0 ± 8.4	57.9 ± 8.3	58.8 ± 9.0	56.3 ± 6.0	56.3 ± 6.0	56.9 ± 5.7	55.1 ± 5.9	Group × Time	0.59
	CrN-High	59.1 ± 9.0	56.7 ± 7.4	57.9 ± 5.8	58.6 ± 3.8	58.0 ± 4.1	56.8 ± 4.7	57.5 ± 3.8		
Systolic BP	PLA	114.8 ± 6.2	113.9 ± 5.6	112.6 ± 4.9	116.5 ± 6.7	116.0 ± 4.8	117.2 ± 8.5	115.5 ± 5.2	Group	0.37
(mm Hg)	CrM	114.6 ± 5.1	112.6 ± 4.4	112.2 ± 4.2	114.9 ± 5.3	112.9 ± 6.1	113.1 ± 5.8	114.9 ± 3.8	Time	0.02
	CrN-Low	115.9 ± 6.1	113.1 ± 4.1	111.9 ± 5.2	113.5 ± 6.9	114.6 ± 6.9	114.62 ± 5.0	112.5 ± 3.5	Group × Time	0.39
	CrN-High	115.4 ± 6.7	114.2 ± 5.5	111.4 ± 6.6	112.5 ± 6.0	113.5 ± 6.2	113.7 ± 9.7	114.2 ± 7.0		
Diastolic BP	PLA	73.1 ± 6.6	71.9 ± 7.5	72.2 ± 6.6	72.3 ± 7.3	73.1 ± 6.6	74.0 ± 5.5	72.3 ± 5.7	Group	0.66
(mm Hg)	CrM	70.3 ± 6.6	71.5 ± 7.8	72.5 ± 4.1	73.9 ± 5.4	72.8 ± 6.0	73.9 ± 5.0	74.5 ± 4.0	Time	0.29
	CrN-Low	72.2 ± 6.5	73.5 ± 5.4	71.9 ± 6.1	75.5 ± 5.4	74.3 ± 6.2	74.5 ± 4.6	73.1 ± 4.1	Group × Time	0.18
	CrN-High	73.1 ± 7.9	77.1 ± 4.8	73.9 ± 6.5	74.5 ± 5.2	73.7 ± 6.1	72.2 ± 7.9	72.9 ± 4.8		
Lipids										
Total-C	PLA	153.8 ± 28.4	156.2 ± 31.1	161.0 ± 31.8	162.5 ± 32.9	164.5 ± 31.2	164.6 ± 36.7	165.0 ± 36.1	Group	0.59
(mg/dl)	CrM	156.8 ± 29.3	157.2 ± 29.3	160.7 ± 34.3	157.2 ± 31.4	159.3 ± 30.4	160.3 ± 32.8	160.2 ± 28.3	Time	0.06
	CrN-Low	154.5 ± 27.6	154.2 ± 28.0	155.7 ± 31.7	156.0 ± 27.8	158.6 ± 28.9	158.5 ± 26.8	159.5 ± 27.9	Group × Time	0.77
	CrN-High	158.3 ± 30.0	163.2 ± 27.0	162.3 ± 32.6	159.9 ± 26.9	163.9 ± 32.6	162.9 ± 26.8	163.5 ± 27.9		
HDL-C	PLA	55.8 ± 15.3	58.2 ± 15.6	58.8 ± 17.4	59.9 ± 16.1	61.0 ± 15.7	61.6 ± 17.0	62.2 ± 17.9	Group	0.29
(mg/dl)	CrM	53.9 ± 17.1	54.1 ± 16.7	55.6 ± 18.4	55.1 ± 17.2	56.0 ± 17.1	56.9 ± 17.6	56.4 ± 18.8	Time	0.001
	CrN-Low	51.0 ± 14.1	50.8 ± 15.6	51.2 ± 15.0	52.1 ± 14.9	53.8 ± 14.5	54.9 ± 15.2	55.3 ± 15.8	Group × Time	0.64
	CrN-High	52.9 ± 17.5	54.7 ± 17.0	54.2 ± 16.8	54.2 ± 17.3	55.4 ± 16.9	55.2 ± 16.3	55.4 ± 17.7		
Total-C/HDL-C	PLA	2.9 ± 0.8	2.8 ± 0.8	2.9 ± 0.8	2.8 ± 0.8	2.8 ± 0.8	2.8 ± 0.8	2.8 ± 0.8	Group	0.40
Ratio	CrM	3.1 ± 1.0	3.1 ± 0.9	3.1 ± 0.9	3.0 ± 0.8	3.0 ± 0.8	3.0 ± 0.8	3.1 ± 0.9	Time	0.09
(mg/dl)	CrN-Low	3.3 ± 1.2	3.3 ± 1.1	3.3 ± 1.0	3.2 ± 1.0	3.2 ± 1.0	3.1 ± 1.0	3.1 ± 1.0	Group × Time	0.62
	CrN-High	3.3 ± 1.4	3.3 ± 1.4	3.3 ± 1.4	3.3 ± 1.5	3.3 ± 1.4	3.3 ± 1.5	3.3 ± 1.5		
LDL-C	PLA	94.0 ± 26.3	96.5 ± 27.6	98.1 ± 28.1	100.1 ± 29.1	102.4 ± 32.6	102.5 ± 32.3	102.2 ± 32.0	Group	0.72
(mg/dl)	CrM	99.1 ± 25.6	99.0 ± 24.1	102.5 ± 27.7	101.1 ± 24.7	102.0 ± 23.7	103.8 ± 26.5	102.5 ± 22.9	Time	0.06
	CrN-Low	97.2 ± 23.0	97.1 ± 22.4	98.8 ± 24.0	100.0 ± 22.4	102.4 ± 23.1	102.6 ± 21.6	104.4 ± 22.5	Group × Time	0.67
	CrN-High	102.7 ± 27.1	105.3 ± 25.8	105.0 ± 29.8	104.1 ± 26.6	101.5 ± 43.7	106.0 ± 27.0	105.4 ± 25.8		
Triglyceride	PLA	79.2 ± 26.2	82.1 ± 27.9	78.8 ± 24.8	73.9 ± 23.5	72.6 ± 21.0	71.8 ± 22.1	71.1 ± 22.6	Group	0.60
(mg/dl)	CrM	90.7 ± 33.1	88.1 ± 34.1	86.6 ± 30.9	76.2 ± 27.3	72.5 ± 25.9	73.7 ± 27.2	68.9 ± 22.6	Time	0.01
	CrN-Low	88.3 ± 36.9	94.5 ± 37.9	90.6 ± 34.7	79.4 ± 25.3	75.4 ± 20.4	73.2 ± 18.3	72.8 ± 17.9	Group × Time	0.66
	CrN-High	79.4 ± 25.6	86.1 ± 34.1	81.8 ± 29.1	73.0 ± 18.3	70.9 ± 19.5	71.9 ± 19.4	70.2 ± 20.3		
Glucose	PLA	95.8 ± 10.3	103.0 ± 10.4	92.1 ± 8.0	92.2 ± 5.5	91.5 ± 4.0	85.9 ± 26.1	91.9 ± 4.3	Group	0.33
(mg/dl)	CrM	96.3 ± 9.9	85.3 ± 27.0	91.4 ± 6.0	89.8 ± 5.1	91.4 ± 5.4	91.6 ± 5.6	91.2 ± 4.9	Time	0.01
	CrN-Low	96.3 ± 5.5	102.6 ± 8.1	94.1 ± 5.9	91.8 ± 6.5	91.2 ± 6.8	92.9 ± 6.7	92.1 ± 6.2	Group × Time	0.12
	CrN-High	94.7 ± 7.5	96.1 ± 6.4	91.4 ± 6.2	91.1 ± 4.0	90.6 ± 5.4	91.3 ± 4.2	83.8 ± 25.3		
Hepatorenal function										
ALP	PLA	7.9 ± 6.5	11.5 ± 8.0	11.9 ± 8.8	13.3 ± 9.1	13.9 ± 9.9	14.3 ± 9.4	16.9 ± 14.1	Group	0.23
(U/L)	CrM	14.7 ± 9.1	15.9 ± 11.7	15.9 ± 10.4	16.8 ± 12.9	20.5 ± 14.4	25.4 ± 15.3	24.6 ± 14.9	Time	0.001
	CrN-Low	12.3 ± 9.3	16.3 ± 10.3	20.3 ± 10.8	18.7 ± 11.5	21.7 ± 10.9	20.6 ± 10.2	22.8 ± 10.5	Group × Time	0.65
	CrN-High	12.8 ± 8.1	17.5 ± 9.5	16.1 ± 8.2	17.2 ± 11.2	16.2 ± 9.7	17.2 ± 15.6	20.1 ± 18.1		
ALT	PLA	19 ± 8.2	19.5 ± 9.1	20.2 ± 9.6	19.6 ± 8.5	20.2 ± 9.7	20.3 ± 9.3	20.2 ± 9.3	Group	0.70
(U/L)	CrM	20.3 ± 7.3	20.5 ± 7.7	21.4 ± 7.8	21.1 ± 8.4	21.1 ± 8.4	21.0 ± 7.9	21.2 ± 8.7	Time	0.001
	CrN-Low	20.1 ± 7.7	20.63 ± 8.4	20.7 ± 8.5	20.8 ± 8.1	21.3 ± 8.2	21.0 ± 8.3	21.1 ± 8.0	Group × Time	0.66
	CrN-High	21.2 ± 8.5	21.3 ± 8.3	21.5 ± 8.7	21.9 ± 8.6	21.1 ± 8.5	22.6 ± 8.8	22.7 ± 8.8		
AST	PLA	24.7 ± 4.9	23.8 ± 5.3	25.1 ± 4.7	25.4 ± 5.8	25.5 ± 5.8	25.2 ± 6.2	25.5 ± 6.1	Group	0.40
(U/L)	CrM	25.9 ± 7.9	26.5 ± 8.8	27.1 ± 8.9	26.0 ± 9.1	26.3 ± 8.8	26.5 ± 8.6	27.0 ± 9.5	Time	0.12
	CrN-Low	24.1 ± 6.1	24.6 ± 6.3	23.9 ± 5.9	24.7 ± 6.1	24.8 ± 5.3	24.3 ± 5.6	24.6 ± 5.4	Group × Time	0.64
	CrN-High	28.0 ± 15.6	28.8 ± 16.3	28.7 ± 16.4	28.8 ± 14.8	29.6 ± 15.2	28.9 ± 14.8	29.8 ± 16.0		
CK	PLA	260 ± 222	264 ± 231	266 ± 219	262 ± 210	259 ± 211	254 ± 200	247 ± 193	Group	0.40
(U/L)	CrM	160 ± 220	262 ± 222	263 ± 212	240 ± 207	249 ± 195	242 ± 189	237 ± 185	Time	0.06
	CrN-Low	265 ± 275	270 ± 273	272 ± 287	267 ± 271	266 ± 273	256 ± 250	255 ± 252	Group × Time	0.51
	CrN-High	490 ± 931	505 ± 958	497 ± 940	475 ± 864	470 ± 863	453 ± 809	470 ± 894		
LDH	PLA	168 ± 40	158 ± 24	170 ± 27	172 ± 26	169 ± 22	171 ± 30	171 ± 27	Group	0.08
(U/L)	CrM	168 ± 21	164 ± 31	168 ± 31	162 ± 23	164 ± 28	165 ± 31	172 ± 29	Time	0.39
	CrN-Low	146 ± 25	165 ± 31	155 ± 19	161 ± 23	160 ± 18	158 ± 22	159 ± 18	Group × Time	0.40
	CrN-High	169 ± 36	179 ± 42	183 ± 40	167 ± 47	196 ± 74	176 ± 32	180 ± 27		
BUN	PLA	14.4 ± 4.8	14.0 ± 4.2	14.0 ± 4.3	13.4 ± 4.5	13.2 ± 4.2	12.8 ± 4.1	12.3 ± 3.8	Group	0.28
(mg/dl)	CrM	15.2 ± 5.0	14.8 ± 4.6	14.6 ± 4.7	13.7 ± 4.5	13.4 ± 4.3	13.1 ± 4.3	12.6 ± 4.0	Time	0.001
	CrN-Low	13.5 ± 4.8	13.0 ± 4.5	12.8 ± 4.5	12.2 ± 4.1	12.1 ± 4.1	11.6 ± 3.8	11.4 ± 3.8	Group × Time	0.76
	CrN-High	14.6 ± 5.1	14.5 ± 5.22	14.1 ± 4.9	13.7 ± 5.1	13.2 ± 4.6	12.9 ± 4.3	12.7 ± 4.3		
Creatinine	PLA	1.00 ± 0.15	0.99 ± 0.16	0.99 ± 0.15	0.98 ± 0.15	0.98 ± 0.15	0.98 ± 0.17	0.95 ± 0.14^d^	Group	0.001
(mg/dl)	CrM	0.99 ± 0.18	1.04 ± 0.17	1.04 ± 0.19	1.01 ± 0.19	0.99 ± 0.17	0.99 ± 0.17	1.00 ± 0.17	Time	0.001
	CrN-Low	1.01 ± 0.19	1.08 ± 0.18	1.09 ± 0.17^a,d^	1.05 ± 0.16^a^	1.05 ± 0.17^a^	1.02 ± 0.18^a^	1.01 ± 1.16	Group × Time	0.001
	CrN-High	1.05 ± 0.14^a^	1.11 ± 0.15^a,b^	1.17 ± 0.14^a,b,c^	1.12 ± 0.16^a,b,c^	1.06 ± 0.16^a^	1.03 ± 0.13^a^	1.02 ± 0.14		
BUN: Creatinine	PLA	14.4 ± 4.2	14.2 ± 4.1	14.3 ± 4.1	13.7 ± 3.9	13.5 ± 3.6	13.1 ± 3.4	12.9 ± 3.2	Group	0.11
(mg/dl)	CrM	15.3 ± 4.3	14.5 ± 4.3	14.2 ± 3.9	13.7 ± 4.1	13.6 ± 4.2	13.4 ± 4.1	12.7 ± 3.5	Time	0.001
	CrN-Low	13.7 ± 5.4	12.2 ± 4.6	11.9 ± 4.6	11.8 ± 4.3	11.8 ± 4.4	11.7 ± 4.1	11.5 ± 4.0	Group × Time	0.08
	CrN-High	14.0 ± 4.8	13.2 ± 4.6	12.0 ± 3.9	12.3 ± 4.0	12.6 ± 3.9	12.5 ± 3.8	12.5 ± 3.9		

Data are mean ± SD. To convert respective values to mmol/L multiply total cholesterol, HDL-C and LDL-C to mmol/L multiply by 0.0259; triglycerides by 0.0113, BUN by 0.357 and glucose by 0.0555. To convert ALP, ALT, AST, CK, LDH, to μkat/L multiply by 0.0167. To convert creatinine to μmol/L multiply by 88.54. *p* < 0.05 is considered significant

*Statistical notations*. ^a^denotes a significant difference from PLA. ^b^denotes a significant difference from CrM. ^c^denotes a significant difference from CrN-Low. ^d^denotes a significant difference from CrM, CrN-Low, and CrN-High

#### Hemodynamic profile

Findings for heart rate (HR), systolic blood pressure (SBP), and diastolic blood pressure (DBP) data are presented in Table 1. Overall, we observed a significant trend for time as systolic blood pressure changed throughout the study so that time 0 was greater than 0.5 h and 1 h post supplementation (*p* < 0.05). Further, the 1 h assessment was significantly lower than SBP at 0, 2, 3, 4, and 5 h post-supplementation (*all*, *p* < 0.01). We did not observe any significant trends for DBP and no between group effects were noted for SBP or DBP at any time point.

### Study 2: chronic supplementation

Seventy-one participants were initially recruited for Study 2 and completed consent forms, and all participated in the required familiarization session. Of the original 71 participants, 48 completed the study. Twenty-three participants dropped out after the familiarization, 22 due to time constraints and one due to apprehension of the muscle biopsy procedure. Fifty-three participants were randomized to the four treatment groups. Twelve participants were initially randomized to the PLA group, but one participant was dropped due to missed scheduled laboratory visits. Fifteen were initially randomized to the CrM group. Four participants dropped out of this group, one due to missed scheduled laboratory visits, one developed abdominal pains after the second day of supplementation, one due to a family emergency, and one withdrew due to time constraints. There were no withdraws from either creatine nitrate (CrN) group.

#### Compliance, side effects, training, and diet

All participants were 100 % compliant with the ingestion of the supplements and 99.4 % were compliant with the proper timing of the supplements.

Our analysis of side effects examining the frequency and severity of dizziness, headache, tachycardia, heart skipping or palpitations, shortness of breath, nervousness, blurred vision, and any other unusual or adverse effects demonstrated no time (*p* = 0.35) or group × time (*p* = 0.34) effects for any variable. However, some participants reported minimal (no greater than 1 in severity) side effects such as dizziness, headache, tachycardia, heart skipping or palpitations, shortness of breath, nervousness, and blurred vision; however, these symptoms where similar amongst the treatment groups over the 28-d period (PLA = 11, CrM = 11, CrN-Low = 13, CrN-High = 13).

#### Dietary characteristics

We observed no significant time effects for daily caloric (absolute: *p* = 0.38; relative: *p* = 0.37), protein (absolute: *p* = 0.74; relative: *p* = 0.77), carbohydrate (absolute: *p* = 0.23; relative: *p* = 0.15), and fat (absolute: *p* = 0.41; relative: *p* = 0.57) intake. There were also no significant group × time effects for daily caloric (absolute: *p* = 0.89; relative: *p* = 0.94), protein (absolute: *p* = 0.40; relative: *p* = 0.39), carbohydrate (absolute: *p* = 0.31; relative: *p* = 0.33), and fat (absolute: *p* = 0.64; relative: *p* = 0.54) intake.

#### Clinical chemistry panels, plasma creatine plasma nitrates, muscle creatine

**Fig. 3:**
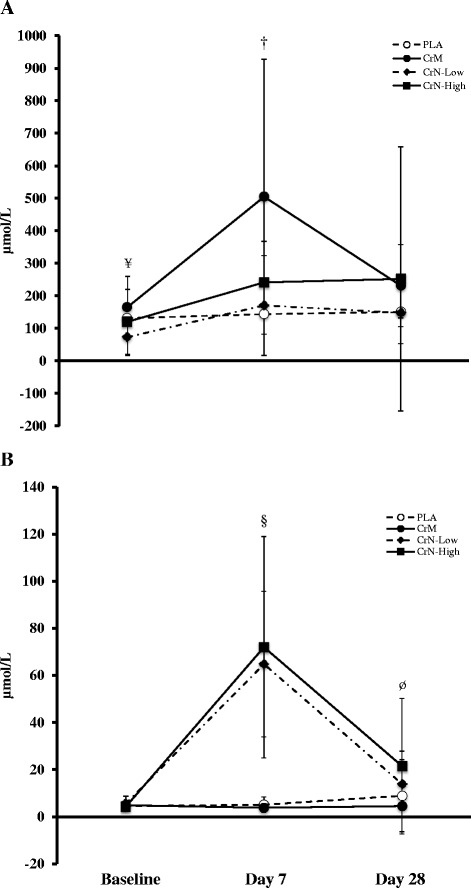
Data represent mean ± SD plasma creatine (**a**) and plasma nitrate (**b**) concentrations following 7 and 28 days supplementation. Statistical notations (*p* < 0.05 considered significant): (¥) denotes CrM significantly greater than CrN-Low. (†) denotes CrM significantly greater than PLA, CrN-Low, and CrN-High. (§) denotes CrN-Low and CrN-High significantly greater than PLA and CrM. (Ø) denotes CrN-High significantly greater than CrM

Results for the plasma lipid, glucose, nitrate and creatine are presented in Table 2 and Fig. 3 while corresponding hepatorenal and blood cell characteristics are presented in Table 3. As a whole, there were no significant time or group × time effects for any blood safety marker. There were significant group, time, and group x time interactions for plasma nitrate and plasma and intramuscular creatine concentrations (*all*, *P* < 0.04). After 7-d of supplementation, there was a significant increase in plasma nitrates in CrN-Low (59.5 μmol/L, 95 % CI 42.6, 76.4) and CrN-High (76.5 μmol/L, 95 % CI 50.7, 84.3) groups, with no concomitant increase observed for the PLA (0.49 μmol/L, 95 % CI −17.7, 18.6) and CrM groups (−1.1 μmol/L, 95 % CI −19.2, 17.1, *p* < 0.001). By d-28, nitrate levels had dropped for the CrN-Low group to a non-significant level relative to baseline (8.52 μmol/L, 95 % CI, −1.9, 18.9), while the CrN-High group remained elevated relative to baseline, but at a concentration ~25 % of the d-7 value (17.09 μmol/L, 95 % CI, 6.7, 27.4). Between group analysis further demonstrated that while the CrN-High condition remained significant relative to the CrM treatment (*P* < 0.03), it no longer remained significant relative to the PLA treatment (Table 2). Table 4 presents the assessment of the blood chemistry changes in serum lipids, glucose, muscle and hepatorenal function associated with supplementation. No significant differences were noted between groups for any analyte.

**Table 2:** Anthropometry and blood lipid, glucose, nitrate, and creatine characteristics of Study 2 participants

		Day				
Variable	Group	Baseline	7	28		p-level
Body Weight	PLA	77.3 ± 11.9	77.6 ± 12.1	77.4 ± 12.7	Group	0.003
(kg)	CrM	81.7 ± 13.2	82.4 ± 13.4	82.6 ± 14.0	Time	0.002
	CrN-Low	72.0 ± 9.7	72.2 ± 9.9	72.7 ± 10.0	Group × Time	0.29
	CrN-High	90.8 ± 13.4	90.8 ± 13.2	92.0 ± 14.3		
Fat Mass	PLA	12.7 ± 6.3	12.6 ± 6.3	12.7 ± 6.3	Group	0.02
(kg)	CrM	12.9 ± 5.3	13.0 ± 5.5	13.2 ± 5.2	Time	0.17
	CrN-Low	8.9 ± 4.7	8.7 ± 4.6	9.1 ± 4.7	Group × Time	0.90
	CrN-High	16.5 ± 6.9	16.2 ± 6.4	16.6 ± 7.4		
Fat-Free Mass	PLA	58.1 ± 8.0	58.5 ± 8.0	58.3 ± 8.3	Group	0.01
(kg)	CrM	62.4 ± 8.7	62.5 ± 8.8	62.9 ± 9.2	Time	0.02
	CrN-Low	56.9 ± 7.4	57.4 ± 7.3	57.3 ± 7.3	Group × Time	0.50
	CrN-High	67.4 ± 8.1	67.7 ± 8.2	68.5 ± 8.2		
Body Fat	PLA	17.8 ± 6.9	17.1 ± 6.7	17.2 ± 6.7	Group	0.07
(%)	CrM	16.7 ± 4.0	16.8 ± 4.3	16.8 ± 3.7	Time	0.24
	CrN-Low	13.1 ± 5.4	12.8 ± 5.2	13.2 ± 5.2	Group × Time	0.78
	CrN-High	19.2 ± 5.9	18.7 ± 5.5	18.8 ± 6.3		
Total Body Water	PLA	51.3 ± 4.5	50.6 ± 5.1	52.5 ± 6.9	Group	0.18
(%)	CrM	51.4 ± 3.3	50.8 ± 3.7	49.4 ± 8.4	Time	0.64
	CrN-Low	52.9 ± 4.6	54.1 ± 5.1	52.7 ± 4.2	Group × Time	0.47
	CrN-High	50.6 ± 6.5	48.8 ± 4.1	49.0 ± 4.7		
Lipids, Glucose, Nitrate, and Creatine						
Total-C	PLA	165.0 ± 33.4	161.5 ± 38.8	164.6 ± 38.8	Group	0.19
(mg/dl)	CrM	174.4 ± 25.5	175.4 ± 27.8	183.4 ± 41.1	Time	0.38
	CrN-Low	151.9 ± 35.8	149.7 ± 29.5	150.0 ± 37.5	Group × Time	0.83
	CrN-High	162.7 ± 25.1	155.9 ± 23.8	160.7 ± 29.1		
HDL-C	PLA	49.2 ± 11.4	50.6 ± 10.2	50.3 ± 12.8	Group	0.62
(mg/dl)	CrM	51.8 ± 15.2	54.7 ± 15.5	55.2 ± 17.4	Time	0.36
	CrN-Low	48.6 ± 15.7	48.3 ± 15.1	47.9 ± 12.8	Group × Time	0.85
	CrN-High	46.7 ± 10.7	47.2 ± 12.3	48.7 ± 10.6		
Total-C/HDL-C	PL	3.45 ± 0.71	3.23 ± 0.64	3.30 ± 0.59	Group	0.82
Ratio	CrM	3.70 ± 1.46	3.50 ± 1.34	3.69 ± 1.58	Time	0.24
(mg/dl)	CrN-Low	3.26 ± 0.81	3.32 ± 1.04	3.29 ± 1.05	Group × Time	0.74
	CrN-High	3.62 ± 0.82	3.47 ± 0.88	3.44 ± 0.94		
LDL-C	PLA	93.4 ± 23.4	91.7 ± 29.6	95.8 ± 34.6	Group	0.34
(mg/dl)	CrM	103.8 ± 33.3	106.6 ± 33.5	112.2 ± 49.0	Time	0.36
	CrN-Low	86.3 ± 22.6	84.8 ± 23.7	87.3 ± 28.7	Group × Time	0.88
	CrN-High	95.2 ± 25.0	93.4 ± 22.9	94.3 ± 28.4		
Triglyceride	PLA	109.1 ± 52.5	98.2 ± 36.9	94.2 ± 19.1	Group	0.63
(mg/dl)	CrM	114.5 ± 60.6	93.1 ± 30.9	112.0 ± 42.6	Time	0.16
	CrN-Low	87.5 ± 43.2	90.7 ± 49.1	85.8 ± 30.7	Group × Time	0.69
	CrN-High	104.8 ± 47.1	85.6 ± 39.2	97.2 ± 49.8		
Glucose	PLA	102.0 ± 18.6	102.1 ± 15.3	94.9 ± 12.6	Group	0.74
(mg/dl)	CrM	97.5 ± 13.9	99.7 ± 15.1	100.4 ± 16.8	Time	0.70
	CrN-Low	96.1 ± 8.5	96.1 ± 7.9	96.4 ± 17.9	Group × Time	0.27
	CrN-High	97.1 ± 9.4	93.2 ± 5.2	96.0 ± 9.4		
Nitrate	PLA	4.5 ± 2.0	5.1 ± 3.3	9.0 ± 15.3	Group	0.001
(μmol/L)	CrM	4.9 ± 3.6	3.8 ± 1.8	4.5 ± 1.8	Time	0.001
	CrN-Low	5.6 ± 3.1	64.8 ± 30.9^a,b^	13.9 ± 13.9	Group × Time	0.001
	CrN-High	4.3 ± 1.6	72.0 ± 47.3^a,b^	21.5 ± 28.9^b^		
Creatine	PLA	131.1 ± 92.2	143.5 ± 131.9	150.6 ± 152.0	Group	0.04
(μmol/L)	CrM	165.2 ± 94.4^c^	504.9 ± 422.8^d^	231.2 ± 126.1	Time	0.001
	CrN-Low	73.0 ± 56.4	170.4 ± 153.7	147.9 ± 95.3	Group × Time	0.01
	CrN-High	120.3 ± 99.8	241.3 ± 131.9	251.9 ± 406.4		

Data are mean ± SD. To convert respective values to mmol/L multiply total cholesterol, HDL-C and LDL-C multiply by 0.0259; triglycerides by 0.0113, BUN by 0.357 and glucose by 0.0555. To convert creatinine to μmol/L multiply by 88.54. *p* < 0.05 considered significant

*Statistical notations*. ^a^denotes a significant difference from PLA. ^b^denotes a significant difference from CrM. ^c^denotes a significant difference from CrN-Low. ^d^denotes a significant difference from PLA, CrN-Low, and CrN-High

**Table 3:** Hepatorenal and blood cell characteristics of Study 2 participants

		Day				
Marker	Group	0	7	28		p-level
Hepatorenal						
ALPS	PLA	11.2 ± 14.6	10.5 ± 10.7	12.4 ± 10.9	Group	0.44
(U/L)	CrM	11.4 ± 9.5	12.0 ± 10.4	11.0 ± 7.8	Time	0.36
	CrN-Low	15.5 ± 11.4	16.2 ± 11.9	19.5 ± 15.9	Group × Time	0.85
	CrN-High	12.0 ± 7.1	13.6 ± 6.1	14.3 ± 11.7		
ALT	PLA	27.2 ± 8.6	30.2 ± 13.8	32.1 ± 30.6	Group	0.24
(U/L)	CrM	28.9 ± 16.6	25.1 ± 10.0	32.7 ± 22.9	Time	0.06
	CrN-Low	21.3 ± 6.7	19.9 ± 4.4	22.4 ± 6.7	Group × Time	0.70
	CrN-High	28.7 ± 13.2	30.0 ± 15.7	36.8 ± 23.2		
AST	PLA	31.2 ± 13.3	30.1 ± 11.6	29.5 ± 13.4	Group	0.90
(U/L)	CrM	28.3 ± 10.2	26.4 ± 7.7	32.5 ± 12.3	Time	0.56
	CrN-Low	26.0 ± 7.0	30.6 ± 25.3	26.0 ± 6.0	Group × Time	0.47
	CrN-High	26.4 ± 5.9	28.7 ± 7.0	32.0 ± 7.6		
CK	PLA	252 ± 128	294 ± 399	206 ± 161	Group	0.79
(U/L)	CrM	285 ± 169	345 ± 319	409 ± 373	Time	0.35
	CrN-Low	283 ± 169	480 ± 987	243 ± 136	Group × Time	0.56
	CrN-High	284 ± 171	349 ± 203	424 ± 287		
LDH	PLA	226 ± 180	167 ± 29	158 ± 34	Group	0.93
(U/L)	CrM	185 ± 62	172 ± 39	184 ± 48	Time	0.20
	CrN-Low	175 ± 50	173 ± 41	166 ± 29	Group × Time	0.23
	CrN-High	173 ± 29	176 ± 30	182 ± 39		
BUN	PLA	16.3 ± 3.6	15.6 ± 5.2	15.7 ± 4.9	Group	0.64
(mg/dl)	CrM	15.3 ± 5.5	15.2 ± 4.9	16.3 ± 6.1	Time	0.48
	CrN-Low	14.9 ± 3.8	13.3 ± 3.9	13.5 ± 4.6	Group × Time	0.64
	CrN-High	14.6 ± 3.2	14.8 ± 2.7	15.7 ± 5.8		
Creatinine	PLA	1.18 ± 0.26	1.19 ± 0.33	1.15 ± 0.27	Group	0.97
(mg/dl)	CrM	1.15 ± 0.46	1.26 ± 0.36	1.23 ± 0.39	Time	0.001
	CrN-Low	1.09 ± 0.25	1.21 ± 0.31	1.23 ± 0.31	Group × Time	0.12
	CrN-High	1.12 ± 0.18	1.15 ± 0.23	1.20 ± 0.23		
BUN: Creatinine	PLA	14.2 ± 3.7	13.9 ± 6.8	13.6 ± 3.1	Group	0.78
(mg/dl)	CrM	13.7 ± 3.6	12.8 ± 5.3	14.0 ± 6.1	Time	0.07
	CrN-Low	14.1 ± 4.2	11.4 ± 3.8	11.3 ± 3.4	Group × Time	0.13
	CrN-High	13.3 ± 3.8	13.3 ± 3.3	13.4 ± 4.8		
Blood cell characteristics						
MCV	PLA	92.2 ± 3.1	91.7 ± 3.7	91.6 ± 3.8	Group	0.79
(fL)	CrM	93.0 ± 3.9	93.7 ± 3.9	93.6 ± 4.7	Time	0.47
	CrN-Low	91.7 ± 3.8	91.8 ± 4.0	92.3 ± 4.2	Group × Time	0.49
	CrN-High	93.3 ± 3.0	93.4 ± 3.1	88.7 ± 17.7		
MCH	PLA	30.4 ± 1.7	30.5 ± 1.3	31.0 ± 2.3	Group	0.45
(pg/cell)	CrM	30.6 ± 1.4	31.2 ± 1.5	31.0 ± 2.6	Time	0.23
	CrN-Low	30.6 ± 1.7	31.6 ± 2.1	31.3 ± 2.2	Group × Time	0.92
	CrN-High	31.2 ± 0.9	31.3 ± 1.4	31.5 ± 1.2		
MCHC	PLA	32.9 ± 1.2	33.3 ± 1.7	33.8 ± 1.8	Group	0.40
(g/dl)	CrM	32.9 ± 0.7	33.3 ± 1.5	33.1 ± 1.9	Time	0.53
	CrN-Low	33.3 ± 0.8	34.5 ± 3.4	33.9 ± 1.7	Group × Time	0.66
	CrN-High	33.5 ± 0.7	33.5 ± 1.6	32.2 ± 6.0		
RBCDW	PLA	13.2 ± 0.5	13.1 ± 0.9	13.1 ± 0.3	Group	0.02
(%)	CrM	13.5 ± 0.9	13.7 ± 0.9	13.9 ± 0.8	Time	0.11
	CrN-Low	13.6 ± 0.6	13.4 ± 0.6	13.5 ± 0.7	Group × Time	0.66
	CrN-High	12.8 ± 0.6	13.2 ± 0.6	13.1 ± 0.4		
Platelet Count	PLA	206 ± 36	188 ± 32	208 ± 70	Group	0.35
(x10^3^/μL)	CrM	234 ± 57	226 ± 91	255 ± 51	Time	0.56
	CrN-Low	218 ± 69	183 ± 50	194 ± 68	Group × Time	0.39
	CrN-High	220 ± 61	412 ± 713	224 ± 59		
WBC	PLA	6.2 ± 1.7	5.3 ± 1.5	6.2 ± 1.1	Group	0.72
(x10^3^/μL)	CrM	6.0 ± 1.7	6.6 ± 1.3	6.4 ± 1.3	Time	0.58
	CrN-Low	6.2 ± 1.6	5.9 ± 1.8	5.4 ± 1.3	Group × Time	0.18
	CrN-High	5.9 ± 1.4	5.5 ± 1.6	6.1 ± 1.6		
RBC	PLA	5.2 ± 0.8	5.01 ± 0.83	4.8 ± 0.6	Group	0.25
(x10^6^/μL)	CrM	5.3 ± 0.6	5.60 ± 0.97	5.1 ± 0.8	Time	0.09
	CrN-Low	5.0 ± 0.5	5.11 ± 0.95	4.9 ± 0.7	Group × Time	0.65
	CrN-High	5.5 ± 0.8	5.08 ± 0.51	5.1 ± 0.5		
Hematocrit	PLA	47.7 ± 7.1	46.0 ± 8.2	44.0 ± 4.5	Group	0.16
(%)	CrM	49.5 ± 6.7	52.5 ± 9.7	47.6 ± 8.4	Time	0.51
	CrN-Low	46.2 ± 5.5	47.1 ± 10.3	44.8 ± 7.1	Group × Time	0.44
	CrN-High	51.2 ± 7.8	47.5 ± 5.2	51.7 ± 15.4		
Hemoglobin	PLA	15.7 ± 2.5	15.3 ± 2.7	14.8 ± 1.2	Group	0.17
(g/dl)	CrM	16.3 ± 2.1	20.1 ± 10.2	15.7 ± 2.8	Time	0.63
	CrN-Low	15.4 ± 2.1	16.1 ± 3.1	15.2 ± 2.3	Group × Time	0.19
	CrN-High	17.2 ± 2.7	15.9 ± 1.4	18.7 ± 9.5		

Data are mean ± SD

**Table 4:** Assessment of blood chemistry changes denoting lipids, glucose, muscle and hepatorenal function concordant to supplementation

			Treatment				
			PLA	CrM	CrN-Low	CrN-High	Chi-Square
	Cholesterol	No Change	0	0	0	0	0.33
		Normal Base; Exceed Day 7	10	8	12	13	
		Normal Base; Exceed Day 28	1	1	0	0	
		Normal Day7; Exceed Day 28	0	2	1	0	
	HDL-C	No Change	6	9	10	10	0.72
		Normal Base; Exceed Day 7	2	1	0	0	
Lipids & glucose		Normal Base; Exceed Day 28	1	0	1	1	
		Normal Day7; Exceed Day 28	2	1	2	2	
	LDL-C	No Change	9	10	13	12	0.30
		Normal Base; Exceed Day 7	0	0	0	0	
		Normal Base; Exceed Day 28	2	1	0	0	
		Normal Day7; Exceed Day 28	0	0	0	1	
	Triglycerides	No Change	11	11	13	13	0.31
		Normal Base; Exceed Day7	0	0	0	0	
		Normal Base; Exceed Day28	0	0	0	0	
		Normal Day7; Exceed Day28	0	0	0	0	
	Glucose	No Change	10	9	11	11	0.56
		Normal Base; Exceed Day 7	1	1	1	0	
		Normal Base; Exceed Day 28	0	1	0	0	
		Normal Day7; Exceed Day 28	0	0	1	2	
	LDH	No Change	9	9	12	11	0.26
		Normal Base; Exceed Day 7	1	0	0	0	
		Normal Base; Exceed Day 28	0	0	0	2	
		Normal Day7; Exceed Day 28	1	2	1	0	
	Creatine	No Change	7	7	10	11	0.75
	Kinase	Normal Base; Exceed Day 7	2	1	1	0	
		Normal Base; Exceed Day 28	0	0	0	0	
		Normal Day7, Exceed Day 28	2	3	2	2	
Muscle	Creatinine	No Change	11	9	11	11	0.32
		Normal Base; Exceed Day 7	0	0	0	0	
		Normal Base; Exceed Day 28	0	1	0	2	
		Normal Day7; Exceed Day 28	0	1	2	0	
Kidney	BUN	No Change	10	8	11	12	0.37
		Normal Base; Exceed Day 7	0	0	1	0	
		Normal Base; Exceed Day 28	0	2	0	0	
		Normal Day7; Exceed Day 28	1	1	1	1	
	ALP	No Change	11	11	13	13	0.32
		Normal Base; Exceed Day 7	0	0	0	0	
		Normal Base; Exceed Day 28	0	0	0	0	
		Normal Day7; Exceed Day 28	0	0	0	0	
Liver	ALT	No Change	11	9	12	11	0.49
		Normal Base; Exceed Day 7	0	0	0	0	
		Normal Base; Exceed Day 28	0	2	1	2	
		Normal Day7; Exceed Day 28	0	0	0	0	
	AST	No Change	8	8	12	8	0.54
		Normal Base; Exceed Day 7	1	0	1	0	
		Normal Base; Exceed Day 28	1	1	0	2	
		Normal Day7; Exceed Day 28	1	2	0	3	

Data are presented as frequency. Statistical significance is detailed from chi-square analyses

There was a significant increase in plasma creatine for the CrM (312.0 μmol/L, 95 % CI 192.1, 432.0), CrN-Low (126.5 μmol/L, 95 % CI, 15.1, 238.0), and CrN-High groups at d-7 (120.9 μmol/L, 95 % CI 14.1, 227.8), all three of which were significant relative to the PLA group (7.7 μmol/L, 95 % CI, −110.0, 131.7, *p* < 0.03). By d-28, only the CrN-High plasma creatine remained significantly elevated relative to baseline (131.5 μmol/L, 95 % CI, 22.5, 240.5) while the CrM (39.0 μmol/L, 95 % CI, −83.4, 161.0) and CrN-Low (103.2 μmol/L, 95 % CI −10.5, 216.9) groups no longer demonstrated a significant elevation. No between group differences at d-28 were otherwise noted.

Intramuscular creatine was significantly increased in the CrM (7.1 mmol/kg DW, 95 % CI, 3.1, 11.0) and CrN-High (4.6 mmol/kg DW, 95 % CI, 0.8, 8.4) groups and significantly decreased in the CrN-Low group (−4.0 mmol/kg DW, 95 % CI −7.7, −0.3) while no significant change was noted for the PLA group (−2.5 mmol/kg DW, 95 % CI, −6.7, 1.7). Between group post-hoc comparisons showed that the CrM and CrN-High groups had significantly greater concentrations relative to the PLA (*p* = 0.002) and CrN-Low groups (*p* = 0.001). On d 28, the CrM group remained the only group to have significantly elevated muscle creatine (8.8 mmol/kg DW, 95 % CI, 5.5, 12.2), while the CrN-High group initial 7-d concentrations abated to a lower concentration (1.4, 95 % CI, −1.8, 4.7 mmol/kg DW). Both the CrN-Low and PLA groups remained relatively unchanged.

#### Anthropometry

While body mass increased over time in all groups by d-28, only the CrM and CrN-High groups had a significant increase in FFM and lean mass, with the CrN-High group significantly greater than the CrN-Low group for both variables (Fig. 5).

#### Bench press performance, anaerobic sprint and Wingate test

**Fig. 4:**
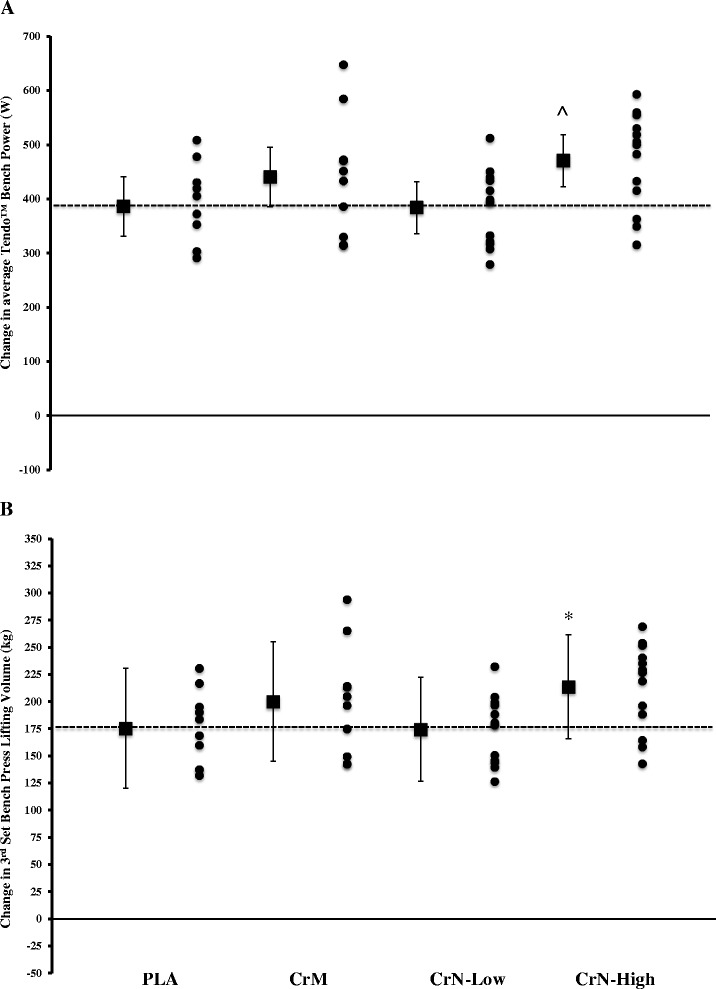
Data represent mean ± 95 % CI change in average Tendo™ bench press power (**a**) and lifting volume based on reps to fatigue during set 3 at 70 % of 1RM bench press (**b**) following 28 days of supplementation. Statistical notations (*p* < 0.05 considered significant): (^) denotes significantly greater than PLA and CrN-Low. (*) denotes significantly greater than PLA

Results for all exercise performance variables are presented in Table 5. Overall, we observed significant time effects and group × time effects for several strength testing variables. Specifically, we observed significant increases in bench press lifting volume (kg) to fatigue for all groups over the 28-d testing period. Only the CrN-High group demonstrated significance differences relative to the PLA group (Fig. 4). Similar findings were noted for peak and average bench press power output as determined by the Tendo™ assessment. Interestingly, post-hoc analyses demonstrated that the CrN-High changes were significantly greater than PLA and CrN-Low (Fig. 4). No significant treatment effects were observed for the Wingate or anaerobic sprint tests.

**Table 5:** Bench press and anaerobic sprint performance characteristics of Study 2 participants

	Group	Day 0	Day 28	Interactions	p-level
Bench press performance					
Max reps	PLA	10.8 ± 2.9	13.0 ± 4.8	Group	0.83
(@70 % 1RM)	CrM	12.0 ± 5.5	15.0 ± 7.1	Time	0.001
	CrN-Low	11.9 ± 2.7	14.0 ± 3.7	Group × Time	0.55
	CrN-High	11.5 ± 3.6	14.8 ± 3.7		
Workload [kg]	PLA	1474.1 ± 373.6	1753.2 ± 548.5	Group	0.17
(wt x reps)	CrM	1827.0 ± 926.3	2255.0 ± 1121.5	Time	0.001
	CrN-Low	1616.5 ± 491.3	1877.3 ± 535.4	Group × Time	0.10
	CrN-High	1927.3 ± 830.2	2516.5 ± 867.2		
Peak power	PLA	425.5 ± 101.1	443.3 ± 107.1	Group	0.10
(W)	CrM	452.9 ± 113.7	521.5 ± 119.9	Time	0.001
	CrN-Low	440.2 ± 76.0	481.1 ± 88.4	Group × Time	0.57
	CrN-High	492.4 ± 95.0	553.3 ± 98.4		
Average power	PLA	357.7 ± 88.4	382.4 ± 93.2	Group	0.03
(W)	CrM	423.8 ± 120.5	456.4 ± 105.0	Time	0.001
	CrN-Low	371.6 ± 70.4	396.4 ± 73.4	Group × Time	0.82
	CrN-High	451.2 ± 91.8	489.4 ± 89.7		
Average velocity	PLA	0.50 ± 0.09	0.53 ± 0.11	Group	0.10
(m/s)	CrM	0.55 ± 0.09	0.62 ± 0.11	Time	0.001
	CrN-Low	0.61 ± 0.83	0.65 ± 0.12	Group × Time	0.74
	CrN-High	0.54 ± 0.11	0.61 ± 0.15		
Anaerobic sprint performance					
Mean power	PLA	668.1 ± 172.0	682.7 ± 142.3	Group	0.03
(W)	CrM	684.2 ± 129.2	720.5 ± 141.5	Time	0.12
	CrN-Low	670.8 ± 124.8	708.9 ± 106.9	Group × Time	0.46
	CrN-High	807.9 ± 91.0	797.7 ± 61.4		
Peak power	PLA	1,464.4 ± 379.7	1,486.8 ± 322.5	Group	0.13
(W)	CrM	1,548.4 ± 351.6	1,611.4 ± 440.5	Time	0.11
	CrN-Low	1,497.4 ± 270.1	1,565.0 ± 278.1	Group × Time	0.95
	CrN-High	1,739.0 ± 275.5	1,783.7 ± 306.8		
Total work	PLA	6,119.6 ± 991.9	6,423.7 ± 896.4	Group	0.55
(J)	CrM	7,188.1 ± 2195.3	6,886.7 ± 744.7	Time	0.76
	CrN-Low	6,760.7 ± 1618.4	6,817.5 ± 1290.8	Group × Time	0.71
	CrN-High	6,716.3 ± 1532.6	6,884.4 ± 1495.3		

Data are mean ± SD

## Discussion and conclusions

**Fig. 5:**
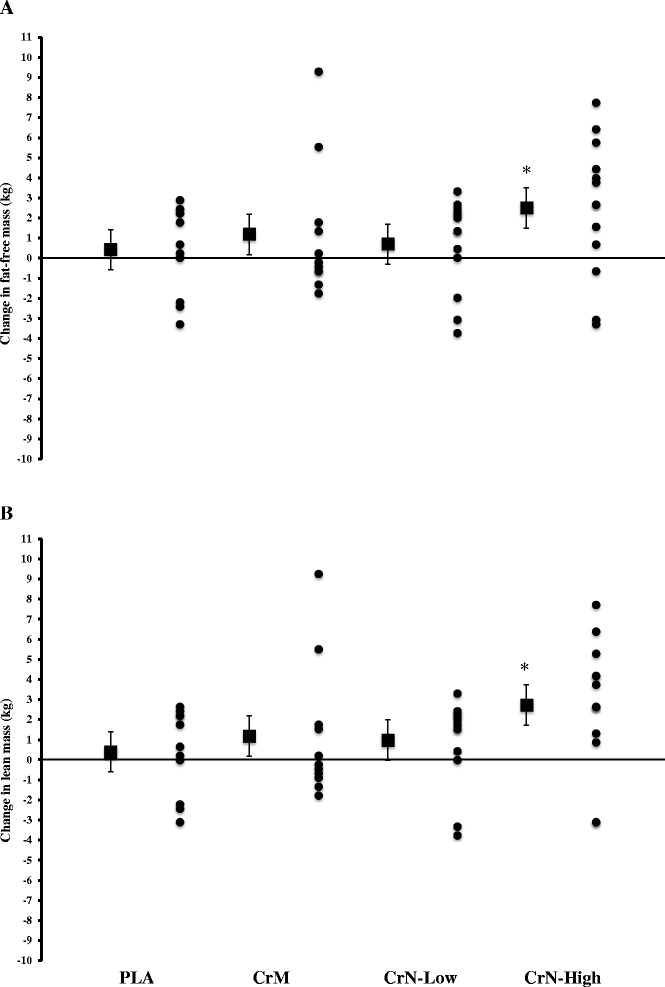
Data represent mean ± 95 % CI change in fat free mass (**a**) and lean body mass (**b**) following 28 days of supplementation. Statistical notations (*p* < 0.05 considered significant): (*) denotes significantly greater than PLA

The primary aim of our studies was to examine the dose-dependent effects of CrN on acute and chronic indices of safety and exercise performance. Accordingly, we performed an acute safety study to assess basic hematologic variables attesting to hepatorenal and muscle enzyme function, blood glucose, lipids, and hemodynamic variables, followed by 28-d of chronic supplementation to examine safety and exercise performance. In Study 1, there were no significant changes in any blood marker or hemodynamic function for any treatment group throughout 5 h of post-ingestion follow-up (Table 1). Study 2 also included a 7-d loading schema. Similar safety findings were observed in Study 2, with two exceptions: ALP and creatine kinase (*see below*). While some side effects were reported, they were distributed amongst all groups, including the PLA group, and were reported to be minimal in regard to severity. There also was a significant increase in several strength parameters for the CrM and CrN-High groups in Study 2. Though the changes associated with CrM and CrN-High were similar, the CrN-High group reached significance when compared to the PLA group for total lifting volume and Tendo™ average power output (Fig. 4). A similar pattern for improvement was also observed for FFM and lean mass (Fig. 5). The differences observed, however, could not be attributed to greater muscle creatine content in the CrN groups compared to CrM. Based on these findings we accept both our hypotheses that CrN is safe when provided up to 3 g/d and is efficacious with regard to changes in strength and body composition.

Several investigations have reported the effects of nitrate supplementation on blood pressure and heart rate [23, 25, 31]. Though we observed a slight trend for a decrease in systolic blood pressure (SBP) (~3 mmHg) in all treatment groups with no significant difference observed among groups. No similar trend was noted for diastolic blood pressure (DBP) or heart rate (HR), nor did we observe any between group differences for the same parameters. Others using beetroot juice as a vehicle for nitrate delivery have reported similar findings. For example, Larsen et al., reported no change in blood pressure following 3-d of ~430 mg nitrate/d [23]. Murphy and colleagues reported similar findings [25]. To the contrary, Webb et al., observed a ~10 mmHg decrease in SBP 2.5 h after ~1,400 mg of nitrate ingestion [31]. In Study 1 we provided 0.5 g nitrate (CrN-Low) and 1 g (CrN-High) and found no evidence that CrN reduced blood pressure or affected HR in comparison to PLA or CrM. Additionally, no participant had a hypotensive response (SBP < 90 mmHg, DBP < 60 mmHg) to either dose of CrN studied. It is therefore conceivable that a dose threshold exists for a single dose of nitrate ingestion as it pertains to hemodynamic alterations. Based on our findings, CrN appears to be well tolerated up to 3 g per serving, yielding 1 g of nitrate, whether ingested acutely or chronically. Adding to these finding is our observation of minimal self-reported side effects associated with acute and chronic CrN ingestion.

During each study we observed a small number of side effects that were rated as minimal in frequency and severity. Collectively, the total number of reported side effects was distributed evenly amongst all treatment groups whereby each treatment group was no different than the PLA group. These findings are similar to previous creatine research investigations. Moreover, creatine supplementation and a diet rich in nitrates have been shown to have a beneficial influence on health [4, 34], either of which may explain the minor, yet positive perturbations of blood lipids observed in Study 1. It should also be noted that changes in lipid fractions have been observed previously accompanying creatine ingestion [9, 26]. There were no consistent, significant effects on any blood variable related hepatorenal or muscle enzyme function. While there were some between group differences, the pattern for these changes was stochastic and inconsistent for any treatment group. For example, there were significant changes across time for high-density lipoprotein (HDL), triglyceride (TG), alkaline phosphatase (ALP), alanine transaminase (ALT), blood urea nitrogen (BUN), BUN-to-creatinine ratio, and glucose. Also, CK values for CrN-High (453 – 505 U/L) were elevated above expected normal ranges (50 – 398 U/L) at baseline and after ingested acutely [28]. However, similar elevations were not observed in CK after 28-d of supplements.

The results of the chronic supplementation trial are intriguing owing to a dichotomy of results suggesting an improvement in exercise performance unmatched by a distinct mechanism of action. While we did observed a significant increase in performance and body composition characteristics generally favoring the CrM and CrN-High groups, these results were not associated with a significant increase in muscle creatine concentrations. In both cases, CrN-High was significantly greater than the PLA group and in the latter example, also greater than the CrN-Low. Despite these differences, only the CrM caused a significant increase in muscle creatine at follow-up.

There was no improvement in cycling anaerobic power output for our assessments despite the CrM group having significantly greater creatine stores than CrN-Low, CrN-High, and PLA groups. It is possible that the dose of CrM used in Study 2 was too low as compared to more typical doses of 5 g. This higher dosing pattern generally yields more consistent results versus studies using 3 g of CrM [5, 14, 27, 32, 33]. These findings contrast claims that there is no need to load when taking CrN due to greater solubility and retention. The effects of nitrate as a precursor to nitric oxide may have also played a role in the development of muscle whereby the acute effects of nitrates increase exercise performance directly, while the chronic effects associated with nitrate supplementation may result from an increase in muscle protein synthesis [6, 13, 30, 35]. While the former point has been demonstrated with endurance exercise, the latter point has recently been hypothesized relative to nitric oxide’s potential role in protein synthesis and is currently under investigation by others [6, 30, 35]. Therefore, it is possible that creatine and nitrate work synergistically, rather than independently, though the mechanism of action is currently unknown.

A strength of this research is that we examined CrN at a supplementation dose that is currently recommended, as well as at a dose twice that, by first performing a general safety assessment, followed by a longer study to monitor chronic safety parameters and exercise performance. A limitation this research is that we did not examine a more typical CrM loading schema using 5 g per serving. We did, however, give this a great deal of consideration and ultimately decided to examine a dose equivalent to those used in the highest CrN group. The decision to use a lower dose partially was based on certain ingredient manufactures assertions that the increased solubility of CrN would increase intramuscular creatine concentrations at a lower dose. This apparently was not the case. However, strength and body composition changes were similar between the CrN-High and CrM groups. It is important to understand that study 2 was not a CrM efficacy study, but rather an assessment of CrN. Future research should examine typically used creatine dosing and/or CrN-High for longer periods of time to better understand the mechanisms of action associated with CrN ingestion. To our knowledge, this is the first study examining the efficacy of CrN in strength training individuals. Although Joy et al. [20] have examined the safety of CrN up to 2 g, we have extended the current body of knowledge to 3 g/d, inclusive of a 7-d loading sequence at four times per day. Creatine nitrate delivered at 3 g was well-tolerated, demonstrated similar performance benefits to 3 g CrM, in addition, within the confines of this study, there were no safety concerns. However, there was no evidence that CrN at recommended or twice recommended doses is more efficacious than CrM at the doses studied.
